# SENNET CONSORTIUM: OPPORTUNITIES AND CHALLENGES

**DOI:** 10.1093/geroni/igad104.1554

**Published:** 2023-12-21

**Authors:** Laura Niedernhofer

**Affiliations:** University of Minnesota, Minneapolis, Minnesota, United States

## Abstract

The Cellular Senescence Network (SenNet) Program was established in 2021 by the NIH Director’s Office Common Fund with the goal of comprehensively identifying and characterizing senescent cells across human lifespan in numerous organs. Mapping senescent cells in mice was added in 2022 to leverage genetic and pharmacologic approaches to drive, report, and ablate senescence in vivo. Senescent cells are implicated in driving most if not all age-related chronic diseases as well as accelerated aging caused by chronic diseases and their treatments (e.g., cancer chemotherapy). Furthermore, therapeutics specifically targeting senescent cells (senotherapeutics) have been developed and are currently being tested in clinical trials for multiple disease and geriatric endpoints. Thus, identifying and characterizing senescent cells throughout human aging and disease states has the potential to significantly impact human health. The SenNet Consortium is a highly collaborative network of scientists across the globe jointly benchmarking tools, technologies, and data analytic approaches, and aligning their activities with other NIH Common Fund initiatives, such as HuBMAP. While mapping senescent cells holds great biomedical promise, there are challenges such as the absence of specific biomarker to identify senescent cells, the appearance of senescence under physiological and pathological conditions, and little knowledge of what drives senescence in vivo. Analogous to the Human Genome Project, to achieve SenNet goals, innovative tools and technologies for single cell and spatial tissue analysis must be developed. Products of SenNet, in addition these new approaches, will include common nomenclature to define senescent cells, novel biomarkers of senescent cells, and publicly accessible databases and 4D atlases of senescent cells in 18 human and murine organs that enables 4D visualization.

